# Cellular Origins of Regenerating Nodules and Malignancy in the FAH Model of Liver Injury after Bone Marrow Cell Transplantation

**DOI:** 10.1155/2016/5791317

**Published:** 2015-12-29

**Authors:** Pei-Rong Wang, Yi Li

**Affiliations:** ^1^Department of Pediatrics, Second Hospital of Shandong University, Jinan 250033, China; ^2^Department of Medicine, University of Washington, Seattle, WA 98195, USA

## Abstract

In previous reports, we and other groups have shown that proliferating hepatocytes are formed by the fusion of donor hematopoietic cells with host hepatocytes in the *Fah^−/−^* model. Thus, it would be interesting to determine whether cell fusion occurs during malignancy. However, it is difficult to demonstrate such processes using this model. Therefore, we established a new strain to study the processes of regenerating nodules and malignancy and their origins. The FAH^−/−^ mouse model was crossed with the ROSAnZ strain and their offspring was genotyped for FAH^−/−^ and ROSAnZ mutations to create a new strain (*Fah^−/−^*-ROSAnZ). Using this strain as recipients, we performed bone marrow transplantation experiments. As a result, we could not demonstrate the presence of any epithelial cells except hepatocytes that were of donor origin in regenerating tissue, and no evidence of cell fusion was found in tumors. The hepatic malignancy was of host origin in these mice. There was higher expression of extracellular matrix proteins and more inflammatory cells in liver tumor nodules than in regenerating normal liver nodules. Hepatocytes generated by fusion with bone marrow cells did not form malignant tumors. Extracellular matrix and inflammatory cells had significantly accumulated in liver tumors.

## 1. Introduction

The liver has the ability to regenerate after certain forms of injury. Understanding these processes in experimental models will help to explain how the liver responds to damage in human hepatic diseases and develop therapeutic strategies for regenerative medicine. One of the most commonly studied models of liver regeneration is the partial hepatectomy model in rodents [1], which demonstrates the capacity of the liver to regenerate after surgical resection. However, the process through which the remnant liver enlarges until the mass of the liver is restored by replication of mature functional cells is not true regeneration because the remnant hepatocytes are not damaged and recruitment of progenitors is not required [2]. Various experimental models have been developed to study liver regeneration processes after a hepatic injury [3, 4]. One of the best experimental systems to study proliferation of hepatocytes is a mouse strain with mutations in the fumarylacetoacetate hydrolase gene (*Fah* ^−/−^) [5]. In this model of hereditary tyrosinemia type I, mice develop fatal liver disease unless maintained on the drug 2-(2-nitro-4-trifluoro-methylbenzyol)-1,3 cyclohexanedione (NTBC) [6]. The hepatic injury in *Fah* ^−/−^ mice can be cured by transplantation of wild-type hepatocytes, resulting in the regeneration of liver nodules expressing donor-derived Fah [7, 8]. However, obtaining human hepatocytes for transplantation is difficult [9, 10]. An interesting finding in this model was obtained by transplantation of hematopoietic cells, which is considered to be a typical example of differentiation plasticity [11]. This plasticity model is particularly robust because the liver nodules contain normal hepatocytes according to both histological and functional criteria, because serum transaminase, bilirubin, and tyrosine levels all normalize after bone marrow cell (BMC) transplantation. Subsequent studies have shown that, rather than being an example of stem cell plasticity, the proliferating hepatocytes are formed by fusion of donor hematopoietic cells with host hepatocytes [12, 13] and that myelomonocytic cells such as macrophages are the responsible cell type [14, 15]. So far, this is the only example in animals and humans of extensive repopulation of damaged livers by cells derived from bone marrow, because the generation of hepatocytes or other solid tissues from BMCs is a very rare event in most experimental models [4]. Such regeneration is due to extensive repopulation of hepatocytes by cell fusion in the FAH model after withdrawal of NTBC, leading to high proliferative pressure. Similar to humans with hereditary tyrosinemia type I [16], *Fah* ^−/−^ mice maintained on NTBC develop hepatocellular carcinoma [6]. Therefore, it would be interesting to determine whether cell fusion occurs in malignancy. However, it is difficult to demonstrate such processes using this model. In a previous report, we established a new transgenic mouse strain (ROSAnZ) that expresses the gene encoding nuclear-localized *β*-galactosidase (*β*-Gal) from the ROSA26 promoter [17], allowing reliable identification of donor-derived cells. Here, we crossed this strain with the FAH strain to generate a new strain (*Fah* ^−/−^-ROSAnZ) and study the processes of regenerating nodules and malignancy as well as their origins.

## 2. Materials and Methods

### 2.1. Generation and Maintenance of Mice

ROSAnZ (C57Bl/6 background) mice were established by gene targeting according to our previous report [17]. *Fah* ^−/−^ mice (C57Bl/6 background) were a kind gift from Markus Grompe. Homozygous *Fah* ^−/−^ and ROSAnZ mice were crossed to obtain heterozygous litters that were bred to establish homozygous *Fah* ^−/−^-ROSAnZ mice. Genotypes were confirmed by PCR analysis of DNA obtained from tail biopsies [5, 18]. *Fah* ^−/−^ and *Fah* ^−/−^-ROSAnZ mice were maintained on NTBC until posttransplantation as described previously [6, 12, 13]. All mice were maintained under specific pathogen-free conditions. Animal experiments were approved by the Institutional Animal Care and Use Committee.

### 2.2. Bone Marrow Transplantation (BMT)

BMCs were harvested from 5-6-week-old female ROSAnZ, C57Bl/6J, or C57BL/6-Tg (CAG-EGFP1Osb/J) mice (Jackson Lab) by flushing their femurs and tibias with sterile phosphate-buffered saline (PBS). Cell suspensions were washed twice in PBS and resuspended in Ca and Mg-free Hank's Balanced Salt Solution with 0.1% bovine serum albumin at 4 × 10^7^ cells/mL. The mice were divided into three groups and irradiated with 1050 cGy from a dual cesium 137 gamma source. Group 1 consisted of *Fah* ^−/−^ mice that received 1.5–2 × 10^7^ ROSAnZ BMCs via tail vein injection. Group 2 was *Fah* ^−/−^-ROSAnZ mice that received 1.5–2 × 10^7^ C57Bl/6J BMCs, and Group 3 was *Fah* ^−/−^-ROSAnZ mice that received 1.5–2 × 10^7^ C57BL/6-Tg (CAG-EGFP1Osb/J) BMCs. NTBC was withdrawn after transplantation and resumed for 1 week intervals after 4, 8, 13, 18, 23, and 27 weeks unless otherwise indicated. Mice were sacrificed at various time points from 1 weeks to 10 months with 5–20 mice/per time after transplantation. Immediately after sacrifice, the liver and other organs were removed for histological analysis. At 2 hours before sacrifice, mice received intraperitoneal injections of bromodeoxyuridine (BrdU) at a dose of 50 *μ*g/g of body weight.

Genomic DNA was isolated and Southern blot analyses were performed by standard techniques. Southern blots were quantified by Phosphorimager analysis (Molecular Dynamics) according to our previous reports [12, 17].

### 2.3. Histochemical Staining for *β*-Gal

Tissues were prepared and stained as described previously [17]. Liver pieces were fixed in 4% paraformaldehyde on ice for 20 minutes, washed with PBS, and then incubated in X-Gal solution for 6–12 hours.

### 2.4. Immunohistochemical Staining of Fah and BrdU

Liver pieces were fixed in Z-Fix Concentrate (Anatech Ltd., Battle Creek, MI) for 24 hours at room temperature (RT), embedded in paraffin, and cut into 3 *μ*m thick sections. The sections were deparaffinized in xylene and dehydrated in graded alcohol concentrations. Sections were treated with 0.3% hydrogen peroxide in methanol for 30 minutes to block endogenous peroxidase activity, incubated in avidin and biotin (Biotin Blocking System, DAKO, Carpinteria, CA) for 10 minutes each at RT, and then incubated sequentially with BLOKHEN II blocking solution (Aves Lab, Tigard, OR) for 20–30 minutes at RT and a chicken anti-mouse Fah antibody (a gift from Markus Grompe) at 4°C overnight. The sections were then incubated with biotinylated goat anti-chicken IgG (H+L) (Vector Laboratories, Burlingame, CA) and then an avidin-biotin-peroxidase complex (ABC/HPR; DAKO) for 45–60 minutes at RT. The color reaction was developed with 3,3′-diaminobenzidine tetrahydrochloride (DAB). Fah^+^ cells exhibited brown-stained cytoplasm after this procedure. To detect BrdU, the sections were then treated with a monoclonal rat anti-BrdU antibody (Abcam, Inc., Cambridge, MA) at 4°C overnight, biotinylated rabbit anti-rat IgG (H+L) (Vector Laboratories) for 45–60 minutes at RT, and then DAB (SK-4100 kit, Vector Laboratories). BrdU^+^ cells exhibited gray-black nuclei after this procedure.

### 2.5. Immunofluorescence Analysis

Tissues were fixed with 4% paraformaldehyde in PBS for 3-4 hours at 4°C, washed with PBS, treated with sucrose, and then frozen in OCT according our previous report [17]. The sections (4 *μ*m thick) were blocked with 10% serum from the same species as the primary antibody for 20–30 minutes at RT and then incubated with polyclonal rabbit or chicken anti-*β*-Gal IgG_2ak_ (ICL Inc., Newberg, OR), chicken anti-mouse Fah, rabbit anti-collagen I, rabbit anti-collagen IV, rat anti-CD45, rabbit anti-CD3 (Abcam, Inc., Cambridge, MA), rat anti-F4/80 (Fitzgerald Industries International, Acton, MA), or monoclonal rat anti-mouse CD45 (BD PharMingen, San Diego, CA) antibodies overnight at 4°C. Sections were incubated with appropriate secondary antibodies including Alexa 594 goat anti-rabbit or chicken IgG (H+L), a FITC-coupled goat anti-chicken antibody (ICL Lab), Alexa Fluor 488 donkey anti-rabbit IgG (H+L) (Molecular Probes, Eugene, OR), or fluorescein rabbit anti-rat IgG (H+L) (Vector Laboratories). After rinsing with PBS, the sections were mounted with 4,6-diaminidino-2-phenylindole (DAPI) and microscopy was performed using a fluorescence microscope (DM IRE2 or DM IRB, Leica, Germany) equipped with a triple bandpass filter to detect emissions at 520 nm for FITC, Alexa Fluor 488, or fluorescein, 610 nm for Alexa 594, and 420 nm for DAPI. Images were pseudocolored using image Openlab 3.1.7 software (Openlab, Germany).

## 3. Results

### 3.1. Regeneration of Nodules by Cell Fusion

**Figure 1:**
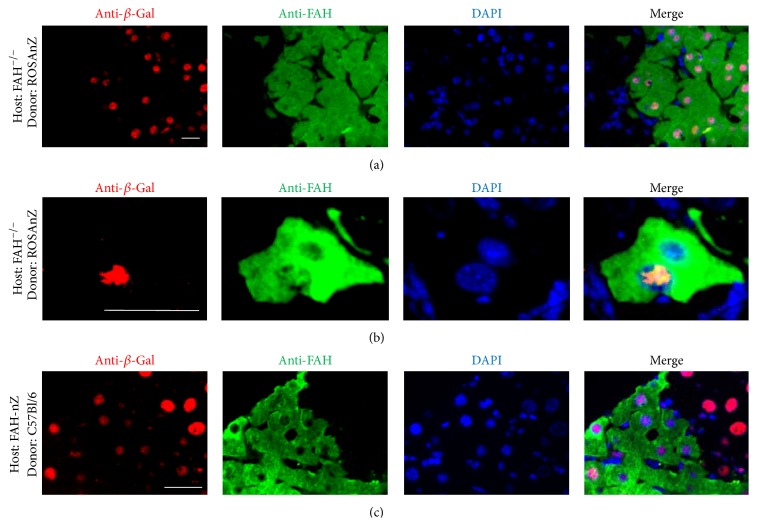
Cell fusion. Colocalization of *β*-Gal (red) and Fah (green) expression in regenerating hepatic nodules (Group 1) (a). Donor-derived hepatocytes with a single Fah^+^ hepatocyte containing two nuclei, of which only one expressed *β*-Gal^+^ in a *Fah* ^−/−^ mouse that received ROSAnZ BMCs (b). Hepatocytes expressing *β*-Gal^+^ in their nuclei, which were formed by host and donor-derived hepatocytes, expressed Fah^+^ in their cytoplasm, which was derived from the donor, in the regenerating nodules of *Fah* ^−/−^-ROSAnZ mice that received C57Bl/6J BMCs (Group 2) (c). Sections were stained with anti-Fah and anti-*β*-Gal antibodies. Scale bar represents 40 *μ*m.

**Figure 2:**
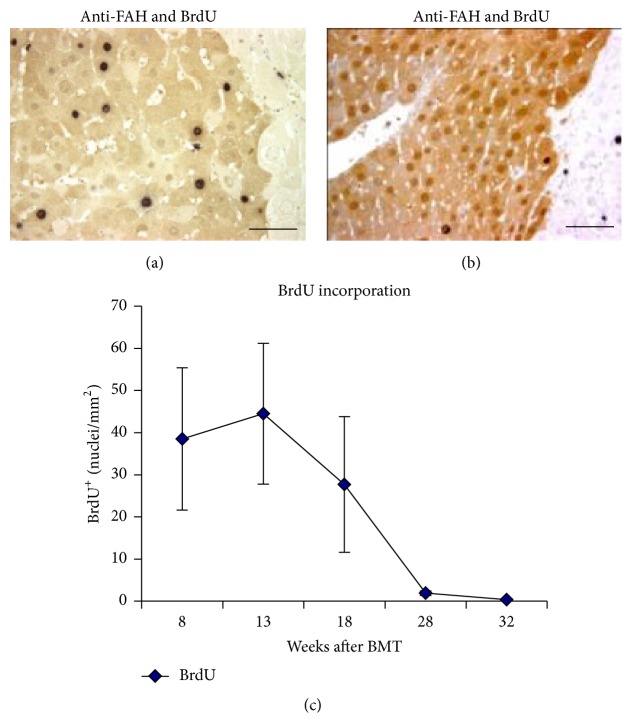
Kinetics of cell proliferation in regenerating nodules double stained for BrdU/FAH by immunohistochemistry. BrdU uptake was detected by an anti-BrdU antibodies staining in regenerating nodules at 13 weeks (a) and 32 weeks (b) after BMT, (c) the number of BrdU^+^ hepatocytes per mm^2^ of the FAH^+^ regenerating nodule surface area (*n* ≥ 5). Means ± SD are shown. Scale bar represents 60 *μ*m.

Mice were sacrificed at various times after transplantation and sections of their livers were stained for *β*-Gal and Fah. Donor-derived hepatocytes expressing Fah in their cytoplasm and *β*-Gal in their nuclei were found in the regenerating nodules of *Fah* ^−/−^ mice that received ROSAnZ BMT (Group 1) (Figure 1(a)). No expression of *β*-Gal in hepatocyte nuclei was observed in host mice. In rare cases, the origin of each nucleus in an isolated Fah^+^ hepatocyte could be determined by immunofluorescence. In Figure 1(b), a single Fah^+^ hepatocyte in a *Fah* ^−/−^ mouse that received ROSAnZ BMT (Group 1) contained two nuclei, but only one was *β*-Gal^+^. This cell must have been the product of a recent fusion event between a host hepatocyte and donor hematopoietic cell, because the nuclear-localized *β*-Gal protein had not yet been redistributed to both nuclei.  Figure 1(c) shows *β*-Gal^+^ hepatocyte nuclei from the host and donor-derived hepatocytes expressing Fah in their cytoplasm in the regenerating nodules of *Fah* ^−/−^-ROSAnZ mice that received C57Bl/6J BMT (Group 2). This is the best model to detect cell fusion in *Fah* ^−/−^-ROSAnZ mice in situ after BMT. DNA levels of donor ROSAnZ engraftment in FAH mice (Group 1) (Figure S1-1 in Supplementary Material available online at http://dx.doi.org/10.1155/2016/5791317) and donor C57Bl/6J engraftment in *Fah* ^−/−^-ROSAnZ mice (Group 2) (Figure S1-2) were detected by Southern blotting. The percentages of donor DNA were 95.63 ± 1.37% and 95.11 ± 3.27% (mean ± standard error of the mean (SEM)) in the bone marrow of recipients and 86.88 ± 7.50% and 92.30 ± 1.31% in the spleens of recipients, respectively. The expansion of these Fah^+^ hepatocytes occurred after NTBC withdrawal by in vivo selection in the presence of dying host hepatocytes. BrdU uptake in regenerating nodules confirmed that hepatocytes cycled less because of coalescence of the regenerating nodules (Figure 2).

### 3.2. Reconstitution of Vascular and Intrahepatic Biliary Architectures in Regenerating Nodules and Their Origins

**Figure 3:**
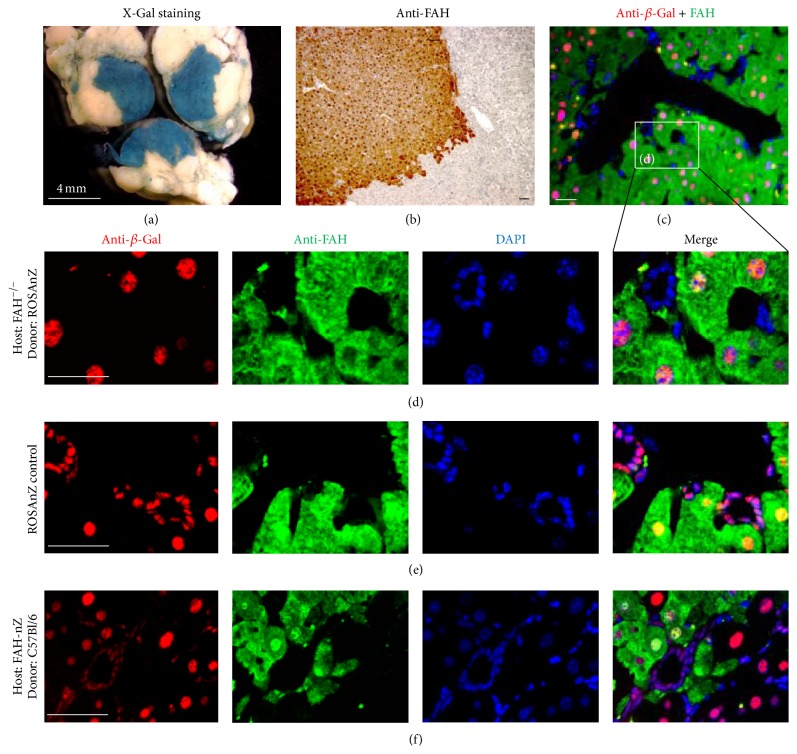
Reconstruction of liver functional units. Strong expression of *β*-Gal was detected in regenerating nodules by whole-mount X-Gal staining of FAH mutant livers (a) and expression of Fah in liver sections by Fah staining (b). Formation of remodeling liver functional units was observed in the regenerating nodules of Group 1 after BMT (c). (d) Higher power view of the indicated region in (d). (e) Intrahepatic biliary architecture in a liver section from a ROSAnZ control mouse. (f) Ductular structures in the regenerating nodules of a *Fah* ^−/−^-ROSAnZ mouse that received C57Bl/6J BMCs (Group 2). Scale bars are 60 *μ*m except for (a) (4 mm).

At 3-4 weeks after transplantation, donor-derived hepatocytes were observed as individual cells or small clusters that later coalesced into large areas of regenerating liver (Figure 3 and Table 1), at which point the liver functions had largely normalized [11–13]. As a regenerating nodule grows, it needs to develop a network of blood vessels and a bile duct system that can provide efficient nutrients, oxygen, and drainage of metabolites by remodeling and reconstitution of vascular and intrahepatic biliary architectures. Although some donor-derived regenerating nodules exceeded 1 mm^2^ at 13 weeks after BMC transplantation, we did not observe any structures of the interlobular bile duct within nodules. Donor-derived hepatocytes proliferated along preexisting blood vessels and interlobular bile ducts of the host and gradually surrounded the interlobular bile duct and blood vessels, which was accompanied by regenerating nodule expansion at ≥15 weeks after BMC transplantation. Central vein and portal triads (containing branches of the hepatic artery, portal vein, and interlobular bile duct) appeared within regenerating nodules and formed normal liver tissue. In regenerating liver tissue from *Fah* ^−/−^ mice after BMT in Group 1, we only observed hepatocytes with nuclei containing *β*-Gal and cells that appeared to be bile duct epithelial (ductular structures) or endothelial cells without *β*-Gal expression in their nuclei (Figures 3(c) and 3(d)). This finding was explored further to identify the origin of these cells in regenerating nodules. In ROSAnZ control mice, virtually every cell nucleus in liver sections contained *β*-Gal, including cells that were not hepatocytes based on their morphology and lacked Fah expression (Figure 3(e)). In the regenerating nodules of *Fah* ^−/−^-ROSAnZ mice after BMT in Group 2 (Figure 3(f)), hepatocyte nuclei as well as nuclei in ductular structures and endothelial cells expressed *β*-Gal, which originated from the host. Thus, we could not demonstrate the presence of other cell types in regenerating liver nodules except for hepatocytes that were of donor origin. These results indicated that the regenerating liver tissues acquired their necessary oxygen and drainage of metabolites from the vascular and interlobular networks of the host during regenerating nodule growth.

### 3.3. Tumor Formation in Transplanted Mice

**Figure 4:**
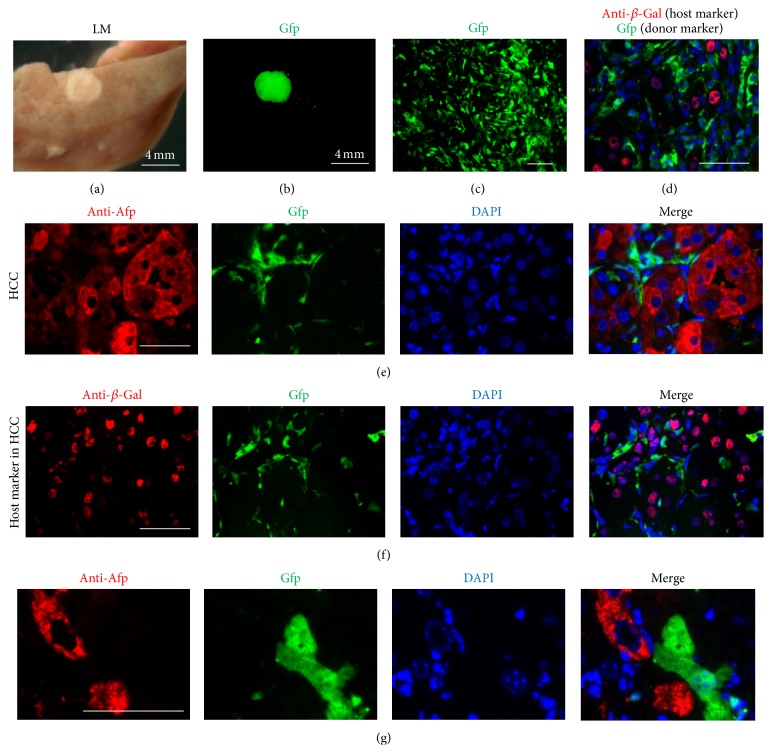
Identification of the cellular origins in liver tumors. Images of gross liver specimens with a tumor (a) and immunofluorescence detection of donor BMC-derived Gfp^+^ nodules (b) in 8-month-old mice of Group 3 as shown in (a) and (b). Expression of Gfp (green) (c) and *β*-Gal (red) (d) was observed in tumor sections. No coexpression of Gfp and anti-*β*-Gal was found in the tumor (e). Serial liver sections were stained with antibodies against Afp (tumor marker; red) (f) and the host marker (*β*-Gal; red). Afp-positive cells confirmed hepatic carcinoma. A higher magnification of the tumor cells stained for Afp revealed that the donor-derived hepatocytes expressing Gfp (green) were negative for Afp (red) (g). Scale bars are 100 *μ*m except for (a) and (b) (4 mm).

**Figure 5:**
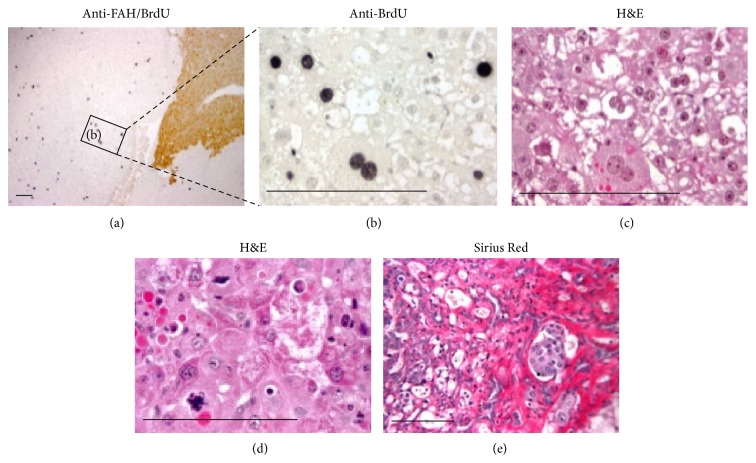
Histochemical characteristic of liver tumors. Liver sections from a 32-week mouse after BMT were stained for Fah (brown) and BrdU (dark nuclei) by immunohistochemistry to indicate the region of Fah^+^ nodules with BrdU uptake (a). The higher power view of the indicated region shows a Fah^−^ tumor with significant BrdU uptake in (b). Sections were also stained with hematoxylin and eosin to identify malignant cells (c and d) and Sirius Red to detect collagen (e). Sections (b) and (c) were serial sections and vascular invasion was also observed in (e). Scale bars are 100 *μ*m.

Similar to humans with hereditary tyrosinemia type I [16], *Fah* ^−/−^ mice maintained on NTBC develop hepatocellular carcinoma [6]. *Fah* ^−/−^ and *Fah* ^−/−^-ROSAnZ mice that survived without NTBC after BMT also developed liver tumors. To determine whether cell fusion occurred in tumors, we used green fluorescence protein (Gfp) as a donor cell marker to observe tumor formation and its origin in *Fah* ^−/−^-ROSAnZ mice that survived without NTBC after BMT in Group 3. The same results were observed in Group 3 in which both donor markers were expressed in the cytoplasm (Gfp) with the host marker (nuclear-localized *β*-Gal protein) in the hepatocyte nuclei of regenerating nodules, which indicated cell fusion. We also detected expression of Gfp in the leukocytes of peripheral blood in Group 3 by flow cytometry. Gfp^+^ cells in recipients were detected by flow cytometry at 1 month and the endpoint after BMT, which were 84.77 ± 10.70% and 87.53 ± 2.90% (mean ± SD), respectively. However, Gfp^+^ nodules were also present in several tumors (Figures 4(a)–4(d)). Despite the presence of these donor markers in tumor nodules, the dysplastic hepatocytes were negative for Gfp expression. We also found that the liver tumor nodules expressed *α*-fetoprotein (Afp) by staining liver sections with an anti-Afp antibody (Figures 4(e)–4(g)). In addition, these liver tumor cells were consistently negative for Gfp and positive for *β*-Gal, which suggested that these liver tumor cells were of host origin, and no evidence of cell fusion was found in these primary tumors. Donor-derived hepatocytes expressing Gfp were not present in Afp^+^ tumor cells of host origin (Figure 4(g)). The liver tumor cells were also consistently negative for Fah expression and cells that were frequently cycling based on BrdU uptake (Figure 5). Histological analysis showed that the nodules were malignant tumors containing abnormal hepatocytes with irregular nuclei, mitotic figures, and giant cells and exhibited a trabecular pattern. In addition, Sirius Red staining revealed collagen in tissue sections and vascular invasion was also observed. These findings demonstrated that the hepatocytes that fused with BMCs do not form malignant tumors in transplanted mice.

### 3.4. Higher Expression of Extracellular Matrix (ECM) Proteins and Increased Inflammatory Cells in Liver Tumor Nodules Compared to Those in Regenerating Normal Liver Nodules

**Figure 6:**
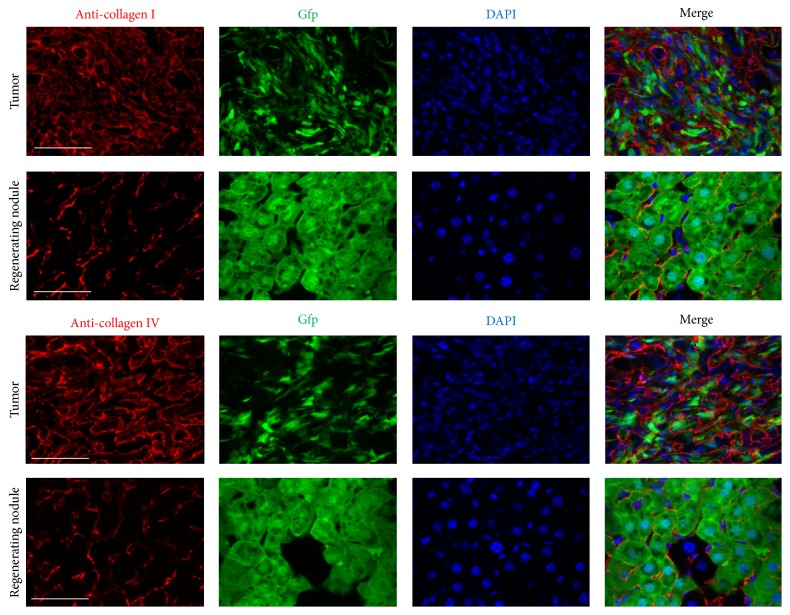
Comparison of ECM protein expression in regenerating nodules and liver tumors. Sections were stained with anti-collagen I and IV antibodies (red). Immunofluorescence was used to detect donor BMC-derived GFP (green). Markedly increased type I and IV collagen deposition was observed in the tumor area compared to that in regenerating liver nodules. Scale bar represents 30 *μ*m.

**Figure 7 fig7:**
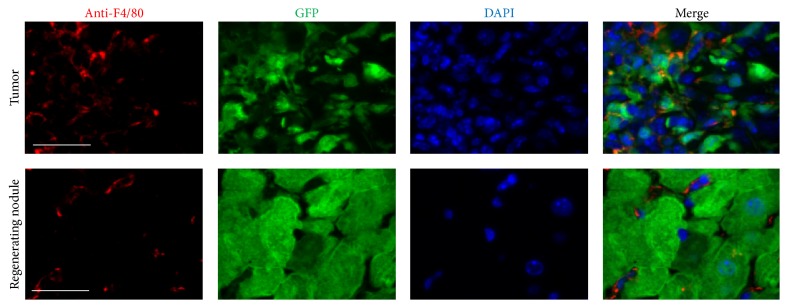
Comparison of macrophages in regenerating nodules and liver tumors. Sections were stained with an anti-F4/80 antibody (red). Immunofluorescence was used to detect donor BMC-derived GFP (green). Markedly increased numbers of macrophages were observed in the tumor area compared to those in regenerating liver nodules. Scale bar represents 60 *μ*m.

**Figure 8:**
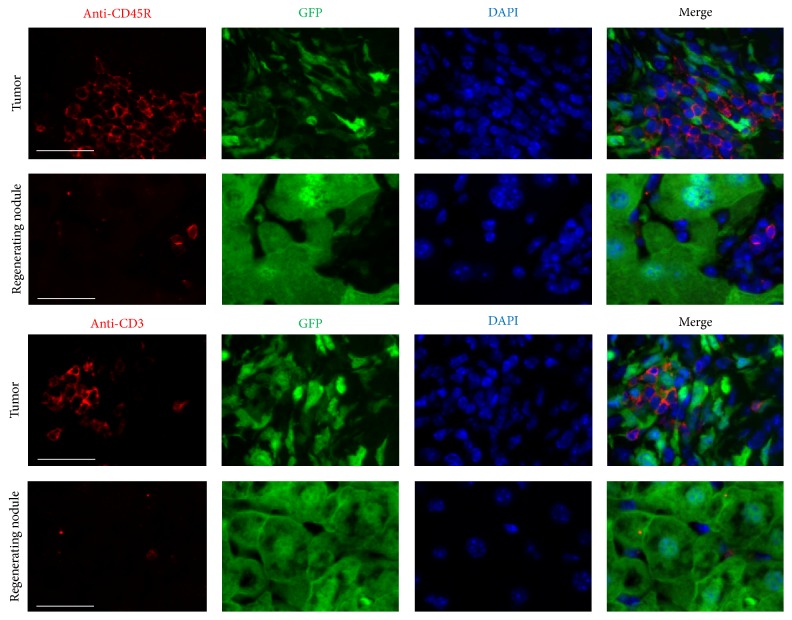
Comparison of B and T lymphocyte infiltration into regenerating nodules and liver tumors. Sections were stained with anti-CD45R and anti-CD3 antibodies (red). Immunofluorescence was used to detect donor BMC-derived GFP (green). Markedly increased numbers of B and T lymphocytes were observed in the tumor area compared to those in regenerating liver nodules. Scale bar represents 60 *μ*m.

We compared ECM protein expression as well as macrophage and T/B lymphocyte infiltration in regenerating and liver tumor nodules (Figures 6 [Fig fig7]–8). Higher expression of ECM proteins and increased numbers of inflammatory cells were observed in liver tumor nodules compared to those in regenerating normal liver nodules. These results indicate that ECM protein expression and macrophage and T/B lymphocyte infiltration were enhanced in liver tumors.

## 4. Discussion

In the *Fah* ^−/−^ model, the regenerating liver nodules are thought to expand from single cells formed by fusion of *Fah* ^−/−^ hepatocytes with *Fah* ^+/+^ donor hematopoietic cells [12, 13]. Single Fah^+^ and *β*-Gal^+^ hepatocytes were observed at 3 weeks after BMT. Increasing cell numbers eventually coalesced and occupied up to 30% of liver tissues, at which point their mitotic rate decreased dramatically (Table 1 and Figure 2). We confirmed that the single cells that initiated nodule formation were formed via cell fusion by identifying donor and host nuclei in the same Fah^+^ and *β*-Gal^+^ hepatocytes in some cases of Group 1. Such cells must represent recently fused cells in which Fah has been redistributed throughout the cytoplasm but *β*-Gal has not yet been newly synthesized and imported into the host nucleus. The strong evidence of cell fusion and its origin were confirmed in our new strain of *Fah* ^−/−^-ROSAnZ mice that received C57Bl/6J BMCs. Both *β*-Gal^+^ hepatocytes from the host and donor-derived hepatocytes expressing Fah in their cytoplasm were observed in the regenerating nodules of Group 2 which is the best model to detect cell fusion in *Fah* ^−/−^-ROSAnZ mice in situ after BMT.

In addition to hepatocytes, several types of cells are present in the liver, including Kupffer, endothelial, and bile duct cells. In the regenerating nodules of *Fah* ^−/−^ mice, Kupffer cells are tissue macrophages derived from donor hematopoietic cells [14, 15]. However, the origins of endothelial and bile duct cells have not been determined. Several studies suggest that new endothelial cells are derived from donor BMCs after transplantation [19–21], including the liver endothelium [22]. Oval cells that generate bile duct cells have also been reported to be derived from bone marrow [23]. However, we did not observe donor-derived endothelia or duct cells in regenerating liver nodules based on staining for nuclear *β*-Gal. Therefore, these cell types presumably proliferated from precursors in surrounding tissues as the nodules expanded.

Cell fusion cured the liver disease of *Fah* ^−/−^ mice, raising the possibility that this approach could ultimately be used clinically. However, a major concern is the oncogenic potential of polyploid cells. It has been reported that tetraploidy precedes aneuploidy in the premalignant condition of Barrett's esophagus [24]. Cytogenetic abnormalities have been found in the hepatocytes of *Fah* ^−/−^ mice cured by BMT [13]. However, abnormalities were also observed in host hepatocytes, and untreated *Fah* ^−/−^ mice developed hepatocellular carcinoma [6]. Thus, the relationship of fusion with malignancy is unclear in this animal model. In our study, we observed carcinomas in the livers of transplanted mice, but the malignant hepatocytes did not express donor markers, even when regenerating and donor-derived liver nodules were present in the same specimens. These findings do not indicate a role of fusion in the malignant transformation of hepatocytes.

Our results revealed higher expression of ECM proteins and the numbers of macrophage and T/B lymphocytes in malignant nodules compared to those in regenerating normal liver nodules, which further supports that ECM proteins and inflammatory cells in tumor microenvironments have many tumor-promoting effects and can aid in the proliferation and survival of malignant cells [25, 26].

## 5. Conclusions

We created a new strain *Fah* ^−/−^-ROSAnZ to study the process of regenerating nodules and malignant formation and their origin. Hepatocytes created by fusion with bone marrow cells did not form malignant tumors and the hepatic malignancy found in these mice was host origins. We could not demonstrate the presence of any epithelial cells except hepatocytes that were of donor origin in regenerating tissue. The extracellular matrix and inflammatory cells were easily recruited to the liver tumors.

## Supplementary Material

Supplemental figure 1: Donor DNA levels in bone marrow, spleens. 1-1: Southern blot analysis of EcoRV-digested genomic DNAs and probed for *ROSAnZ* sequences from mice *ROSAnZ* (Donor) and *Fah*-/- (recipients) genotypes in Group 1. Percentage of donor DNA levels in bone marrow of donors (A), bone marrow of recipients (B), spleen of donors (C) and spleens of recipients (D). The value presents as mean ± SEM. 1-2: Southern blot analysis of EcoRV-digested genomic DNAs and probed for *Fah* sequences from mice with wild-type C57Bl/6 (Donor) and *Fah*-/- - *ROSAnZ* (recipients) genotypes in Group 2. Percentage of donor DNA levels in bone marrow of donors (A), bone marrow of recipients (B), spleen of donors (C) and spleens of recipients (D). The value presents as mean ± SEM, (n ≥20).

## Figures and Tables

**Table 1:** 

Weeks after BMT	Weeks off NTBC	Maximum # of cells per nodule or % regenerating liver	Density of BrdU^+^ cells in Fah^+^ regenerating nodule (mm^2^)
1	1	0	
2	2	0	
3	3	1	
4	3	4	
5	4	5	
6	5	10	
7	6	15	
8	7	20	38.5 ± 16.9
10	8	50	
13	11	3–5%	44.5 ± 16.7
18	15	15–25%	27.7 ± 16.1
23	19	20–30%	
28	23	15–30%	1.89 ± 0.51
32	27	15–30%	0.39 ± 0.23

Donor-derived cells repopulate the livers of transplanted mice in Group 1. The value is presented as mean ± SD (*n* ≥ 5).
