# Supplementary material for: Cellular Origins of Regenerating Nodules and Malignancy in the FAH Model of Liver Injury after Bone Marrow Cell Transplantation
Source: Stem Cells Int. 2015 Dec 29;2016:5791317. doi: 10.1155/2016/5791317 (PMC4709791; doi:10.1155/2016/5791317)
Supplement: Supplementary file 1 — Supplemental figure 1: Donor DNA levels in bone marrow, spleens. 1-1: Southern blot analysis of EcoRV-digested genomic DNAs and probed for ROSAnZ sequences from mice ROSAnZ (Donor) and Fah-/- (recipients) genotypes in Group 1. Percentage of donor DNA levels in bone marrow of donors (A), bone marrow of recipients (B), spleen of donors (C) and spleens of recipients (D). The value presents as mean ± SEM. 1-2: Southern blot analysis of EcoRV-digested genomic DNAs and probed for Fah sequences from mice with wild-type C57Bl/6 (Donor) and Fah-/- - ROSAnZ (recipients) genotypes in Group 2. Percentage of donor DNA levels in bone marrow of donors (A), bone marrow of recipients (B), spleen of donors (C) and spleens of recipients (D). The value presents as mean ± SEM, (n ≥20). [file 5791317.f1.pdf]

Supplemental figure 1

Figure 1-1

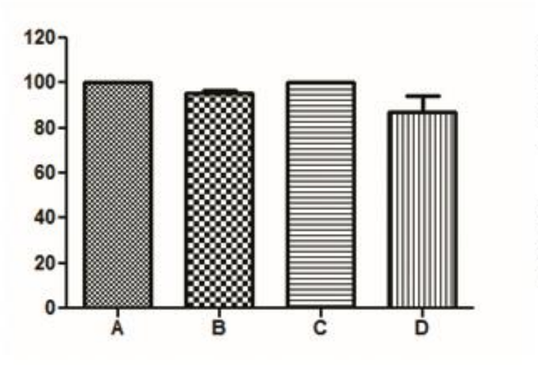

Figure 1-2

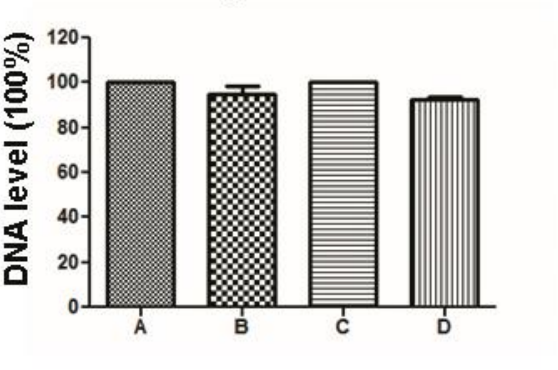

Supplemental figure legends

Supplemental figure 1. Donor DNA levels in bone marrow, spleens. 1-1: Southern blot analysis of EcoRV-digested genomic DNAs and probed for *ROSAnZ* sequences from mice ROSAnZ (Donor) and *Fah-/-* (recipients) genotypes in Group 1. Percentage of donor DNA levels in bone marrow of donors (A), bone marrow of recipients (B), spleen of donors (C) and spleens of recipients (D). The value presents as mean ± SEM. 1-2: Southern blot analysis of EcoRV-digested genomic DNAs and probed for *Fah* sequences from mice with wild-type C57Bl/6 (Donor) and *Fah-/-* - *ROSAnZ* (recipients) genotypes in Group 2. Percentage of donor DNA levels in bone marrow of donors (A), bone marrow of recipients (B), spleen of donors (C) and spleens of recipients (D). The value presents as mean ± SEM, (n ≥20).
